# First person – Phillipe O'Brien

**DOI:** 10.1242/dmm.038323

**Published:** 2018-12-18

## Abstract

First Person is a series of interviews with the first authors of a selection of papers published in Disease Models & Mechanisms (DMM), helping early-career researchers promote themselves alongside their papers. Phillipe O'Brien is first author on ‘Juvenile murine models of prediabetes and type 2 diabetes develop neuropathy’, published in DMM. Phillipe is a Postdoctoral Researcher in the lab of Dr Eva Feldman at the University of Michigan, Ann Arbor, MI, USA, investigating the use of mouse models of disease to understand the development of peripheral neuropathy, a common complication of obesity, prediabetes and type II diabetes.


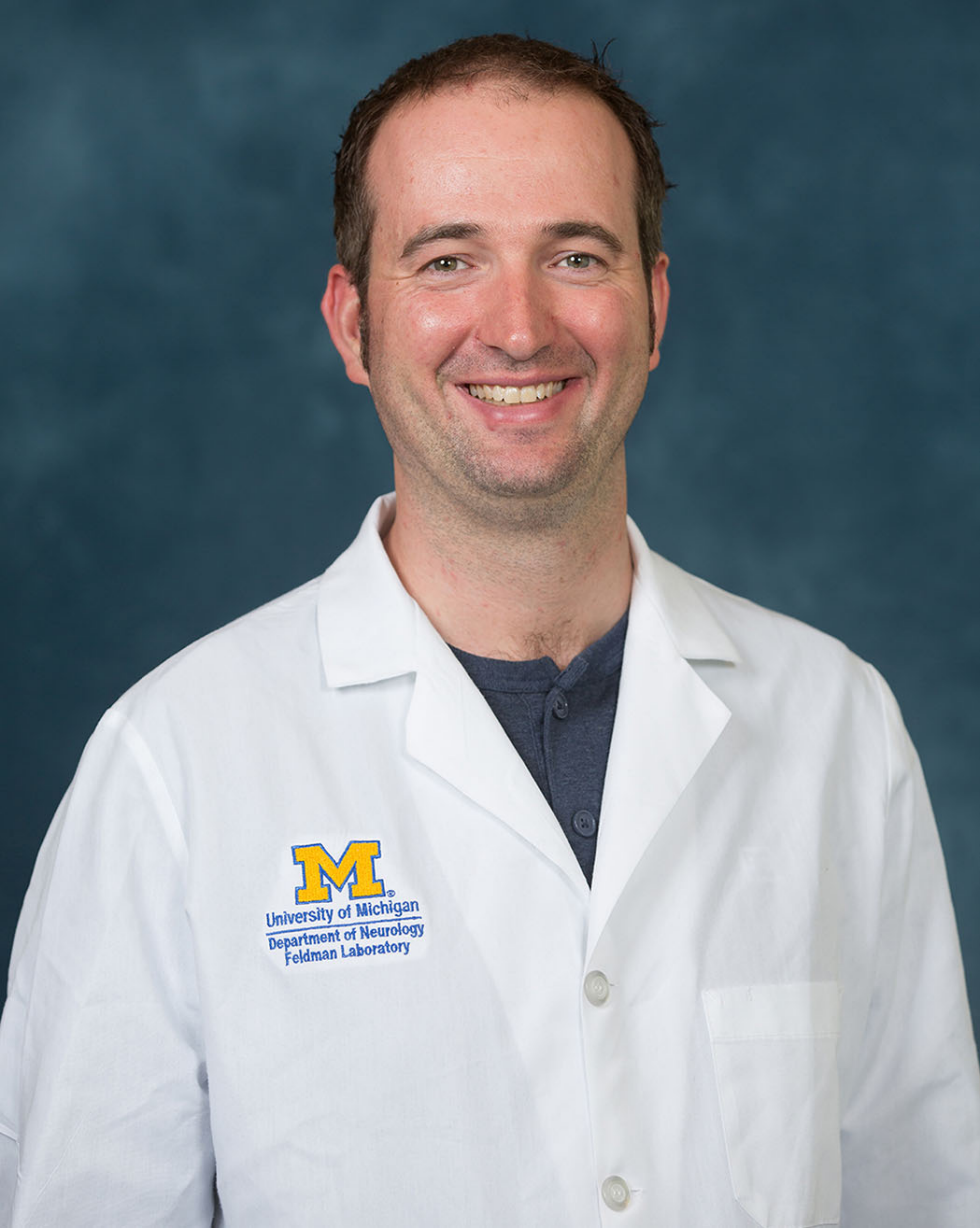


**Phillipe O'Brien**

**How would you explain the main findings of your paper to non-scientific family and friends?**

Obesity, prediabetes and type 2 diabetes are increasing at an alarming rate and each are associated with the development of complications that reduce the patient's quality of life. One of the most common complications is peripheral neuropathy, a disease that affects peripheral nerves, which initially is characterized by the development of painful symptoms but eventually leads to loss of sensation that puts patients at increased risk of injury. A recent study from our group has shown that the prevalence of neuropathy is increasing among children and adolescents with obesity and prediabetes; however, little is known about the mechanisms underlying nerve injury in this vulnerable population. In this study, we identified new mouse models of juvenile prediabetes and type 2 diabetes. Our prediabetic model consists of feeding mice a high-fat diet at a young age while our type 2 diabetic model involves an additional insult, promoting high plasma glucose, a hallmark of type 2 diabetes. These models are more appropriate when exploring the drivers of disease in the youth so that therapeutic strategies can be developed.


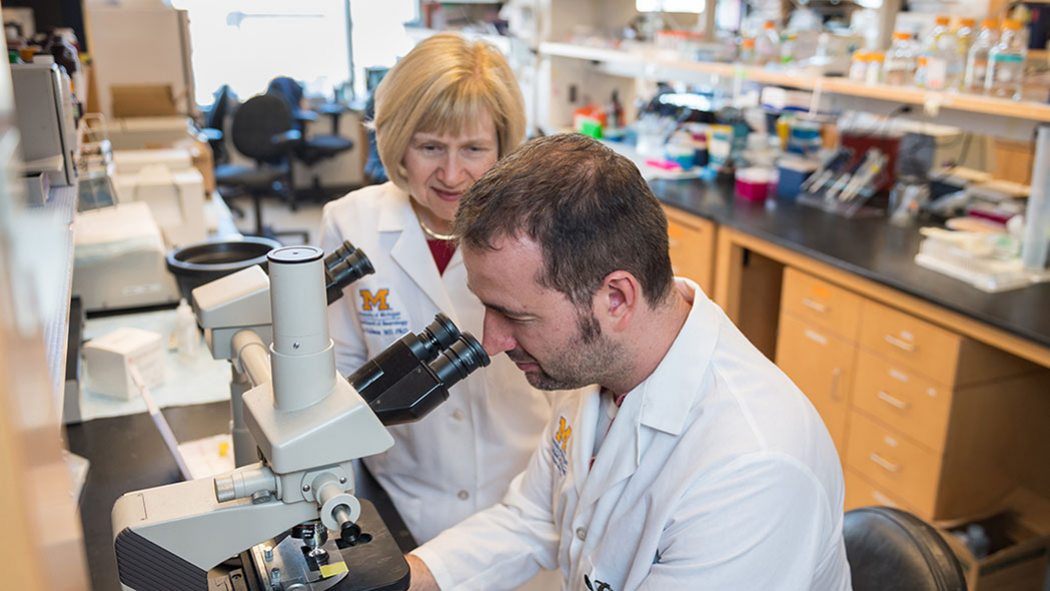


**Phillipe O'Brien with Dr Eva Feldman**

“As juvenile-onset neuropathy is largely understudied, these mouse models are a valuable tool when investigating neuronal changes in the onset and progression of neuropathy in youth.”

**What are the potential implications of these results for your field of research?**

As juvenile-onset neuropathy is largely understudied, these mouse models are a valuable tool when investigating neuronal changes in the onset and progression of neuropathy in youth. Another interesting finding was that we saw no increase in neuropathy severity in our model of type 2 diabetes when compared to our prediabetic model. This suggests that factors other than glucose are involved in disease progression. Recent studies point to dyslipidemia (altered lipid homeostasis) as being an important factor in neuropathy development and our current research efforts are focusing on looking at the role of lipids in disease progression.

**Figure:**
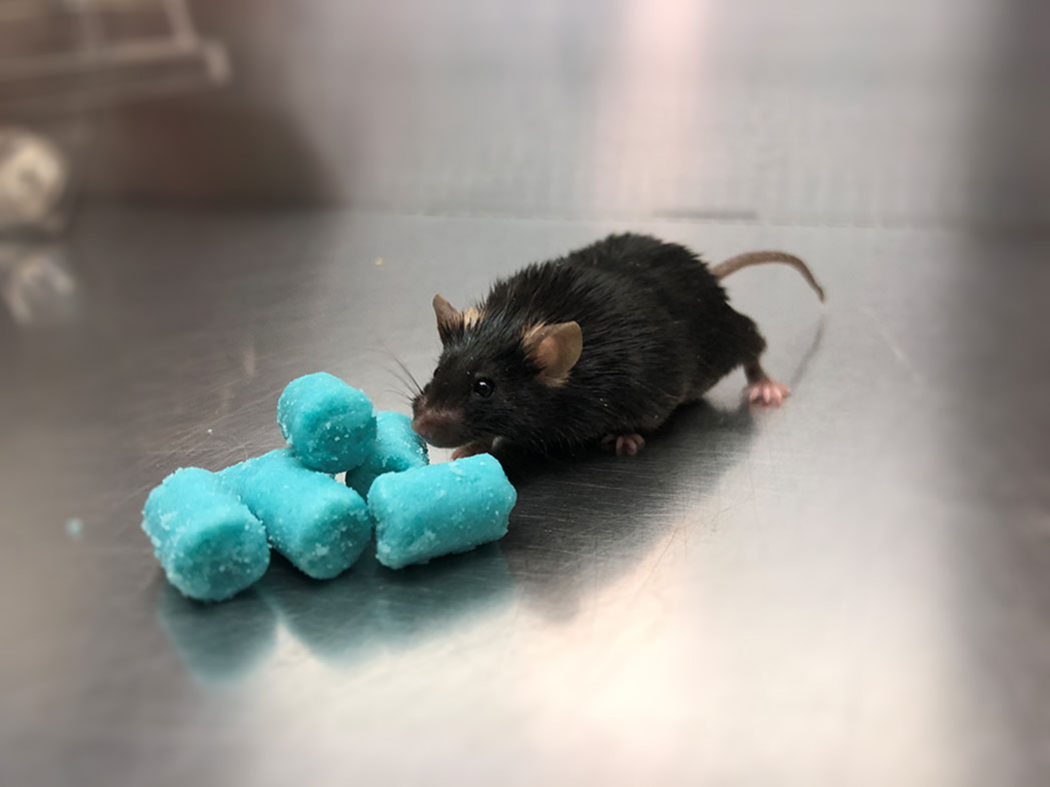
**Male C57BL/6J mice were fed a high-fat diet to induce diet-induced obesity (DIO), a model of prediabetes.**

**What are the main advantages and drawbacks of the model system you have used as it relates to the disease you are investigating?**

One of the main advantages is that both the prediabetic and type 2 diabetic mouse models exhibit a robust, consistent and reproducible neuropathy, something that is not always achievable in other diabetic models. An advantage of using a high-fat diet to induce the desired phenotype is that the time of onset and duration of prediabetes/diabetes is inducible and therefore we can accurately predict neuropathy development. Both models also develop other complications of prediabetes and type 2 diabetes found in patients, including fatty liver disease, diabetic kidney disease and cognitive impairment. Having a model that mimics multi-organ dysfunction as a consequence of prediabetes/diabetes can be quite useful when testing whether therapies have multiple targets.

In order to develop a non-genetic mouse model of type 2 diabetes, a low dose of streptozotocin is administered to animals fed a high-fat diet. This chemotoxic agent selectively targets pancreatic cells to reduce insulin production; without insulin, plasma glucose cannot be cleared, resulting in hyperglycemia, a feature of type 2 diabetes. However, type 2 diabetes is associated with high plasma insulin and so such a model may not be ideal for investigators studying the role of insulin signaling in peripheral neuropathy.

**What has surprised you the most while conducting your research?**

Correcting hyperglycemia in type 1 diabetes can restore peripheral nerve function, strengthening the hypothesis that high glucose is a driver of neuropathy. For this reason, we expected that our model of type 2 diabetes that exhibits high glucose would have more severe (or earlier onset of) neuropathy than our prediabetic model. However, despite the type 2 model having a greater degree of hyperglycemia, we observed no differences in peripheral neuropathy between models. These data suggest that factors other than glucose are involved in type 2 diabetic neuropathy.

**Describe what you think is the most significant challenge impacting your research at this time and how will this be addressed over the next 10 years?**

One of the main challenges in driving peripheral neuropathy research forward is that researchers tend to use different research protocols for developing models of disease. For example, groups use different animal strains, source animals and diets from different vendors and vary the timing of feeding. All of these variables make it difficult to make meaningful comparisons. For this reason, having a consensus among research groups is important to drive research forward.

“Having a consensus among research groups is important to drive research forward.”

**What changes do you think could improve the professional lives of early-career scientists?**

To have a successful career in science you need to be able to manage your time efficiently. From my experience, too often there are non-research-related tasks that are quite distracting. Such distractions should be minimized to allow scientists to make advancements in their studies, unhindered. Having more administrative support would help to reduce such inconveniences.

**What's next for you?**

The findings from this study implicate dyslipidemia in neuropathy development. As the role of lipids in peripheral nerve disease is understudied, the next step is to examine the whole nerve lipidome to identify what lipid species are altered. Following this, our studies will attempt to understand what specific lipid-related mechanisms contribute to disease so that specific therapeutic targets can be identified for the treatment of neuropathy.
